# Role of Fungi in Tumorigenesis: Promises and Challenges

**DOI:** 10.1146/annurev-pathmechdis-111523-023524

**Published:** 2025-01

**Authors:** Silvia Guglietta, Xin Li, Deepak Saxena

**Affiliations:** 1 Department of Regenerative Medicine and Cell Biology, Medical University of South Carolina, Charleston, South Carolina, USA; 2 Hollings Cancer Center, Charleston, South Carolina, USA; 3 Department of Molecular Pathobiology, NYU College of Dentistry, New York, NY, USA; 4 Laura and Isaac Perlmutter Cancer Center, NYU Grossman School of Medicine, New York, NY, USA; 5 Department of Urology, NYU Grossman School of Medicine, New York, NY, USA; 6 Department of Surgery, NYU Grossman School of Medicine, New York, NY, USA

**Keywords:** mycobiome, fungi, microbiome, pancreatic cancer, colorectal cancer, immune response, inflammation, bacteria–fungi interaction, sequencing, bioinformatics tools

## Abstract

The mycobiome plays a key role in the host immune responses in homeostasis and inflammation. Recent studies suggest that an imbalance in the gut’s fungi contributes to chronic, noninfectious diseases such as obesity, metabolic disorders, and cancers. Pathogenic fungi can colonize specific organs, and the gut mycobiome has been linked to the development and progression of various cancers, including colorectal, breast, head and neck, and pancreatic cancers. Some fungal species can promote tumorigenesis by triggering the complement system. However, in immunocompromised patients, fungi can also inhibit this activation and establish life-threatening infections. Interestingly, the interaction of the fungi and bacteria can also induce unique host immune responses. Recent breakthroughs and advancements in high-throughput sequencing of the gut and tumor mycobiomes are highlighting novel diagnostic and therapeutic opportunities for cancer. We discuss the latest developments in the field of cancer and the mycobiome and the potential benefits and challenges of antifungal therapies.

## INTRODUCTION

**Figure 1:**
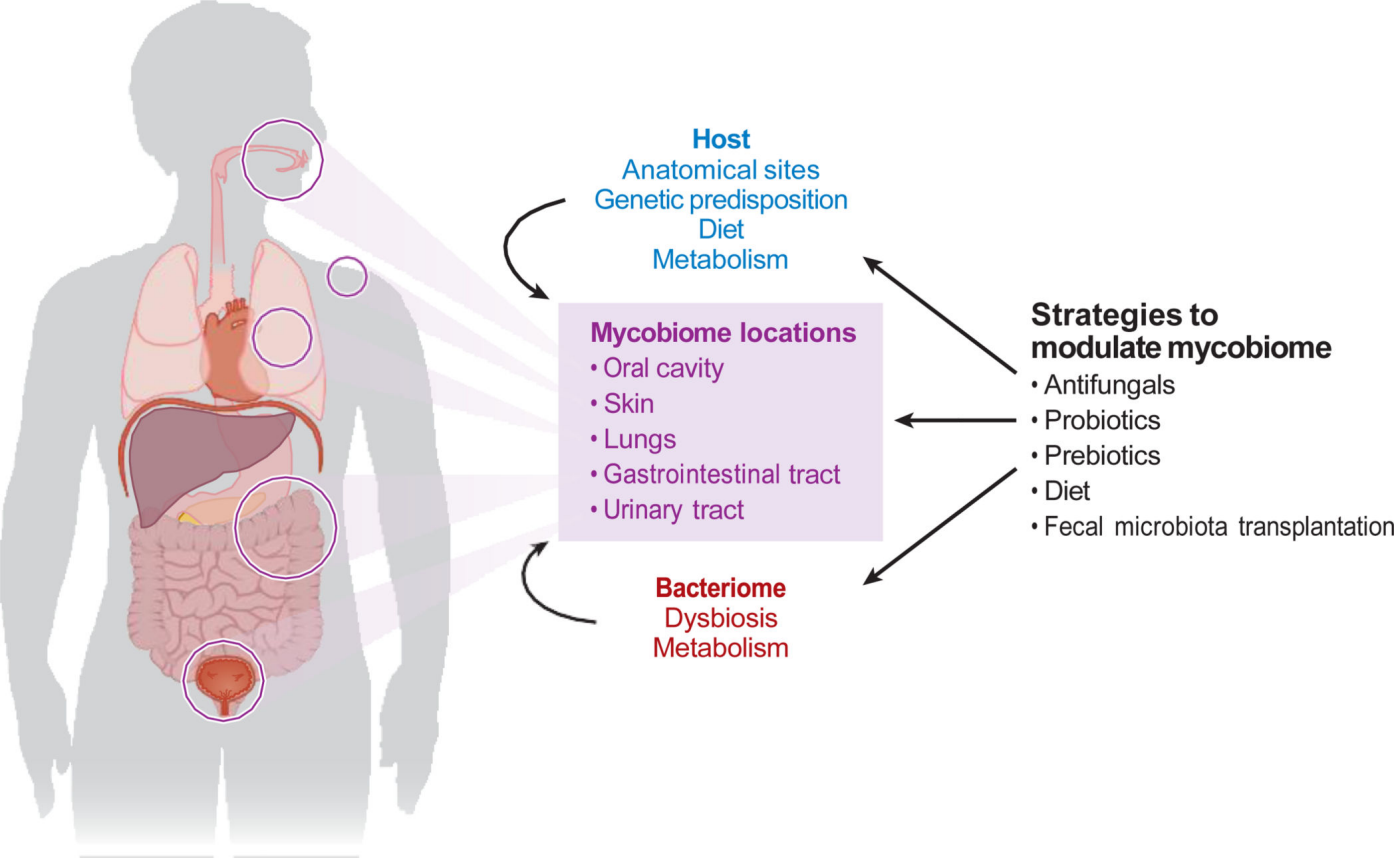
The human mycobiome—an overview. Factors affecting the mycobiome and strategies to modulate the mycobiome are shown.

The human body houses a diverse range of complex microorganisms, including fungi. These fungal communities, collectively referred to as the human mycobiome, make up a part of the greater microbiome, along with the virome and bacteriome (Figure 1) (1–3). The microbiome, in its entirety, has been linked to various aspects of health and disease (4, 5). The mycobiome represents less than 1% of all microbes found in humans and less than 0.1% of those in the gut (1–4, 6–9). Conventional culture methods inform most of our knowledge about fungi. Previously, it was thought that the human body’s mycobiome members were simply commensals, meaning they may not directly contribute to disease (3, 4, 10). However, advancements in sequencing techniques have allowed a more detailed understanding of fungal diversity and its role in human health. This includes both beneficial and harmful interactions between the fungal, bacterial, and viral members of the microbiome and the host’s immune system in the context of health and disease (Figure 1) (3, 11, 12).

The gut mycobiome shows higher variability between individuals and more instability over time compared with the gut bacterial microbiome (4). The gut mycobiome constantly shifts within an individual, both spatially and temporally. Approximately 26 fungal taxa have been identified within the gastrointestinal tract. These include *Saccharomyces*, Ascomycota, Basidiomycota, *Cladosporium*, *Penicillium*, *Trichosporon*, *Aspergillus*, *Candida*, *Malassezia*, and *Debaryomyces* (10, 11). These changes are influenced by various factors such as the individual’s genetic makeup, diet, medication use, lifestyle, and environment (13, 14). In healthy infants the gut mycobiome is initially dominated by Saccharomycetales and *Malassezia* species, which gradually decline and become un-detectable after the first 5 months of age (15, 16). During the first 1–2 years of age, coincident with a dietary shift from breast milk to solid food, the gut mycobiome becomes dominated by *Saccharomyces cerevisiae*, *Cystofilobasidium* spp., Ascomycota spp., and *Monographella* spp., which indicates the prominent role of diet in shaping the gut mycobiome (15). Further compositional changes are noted during adulthood, when the fungal community in healthy individuals shows a substantial increase in gut mycobiome diversity and becomes dominated by the phyla Ascomycota, Basidiomycota, and Zygomycota (4, 11).

The discovery of many common fungal species across the general population suggests the existence of a core gut mycobiome (1–3, 9, 10, 17). The most common taxa discovered in the human gut happen to be Ascomycota and Basidiomycota (18). Also, an individual’s sex and age may impact the relative quantities of fungal endosymbionts in the human gut (1, 3–5, 8, 19). For instance, *Aspergillus* and Tremellomycetes species are more commonly found in males, whereas *Candida* species are more prevalent in females (14). Furthermore, infants are more likely than adults to harbor *Penicillium*, *Aspergillus*, and Tremellomycetes. It is also noteworthy that fungal diversity decreases with age (14).

Recent studies have found links between the gut mycobiome and the progression of various types of cancer, including colorectal (20), pancreatic (21, 22), and head and neck (23) cancers. These insights pave the way for a deeper understanding of cancer pathogenesis and potential new avenues for cancer therapy (24–26). Research indicates that fungal species are present in the tumor microenvironment of most major human cancers, albeit less abundantly than bacteria (12, 26–28).

There is sufficient evidence to suggest that specific microbial signatures are associated with certain types of cancer, reinforcing the notion that the mycobiome can influence cancer pathogenesis (see the sidebar titled Emerging Mechanisms by Which the Mycobiome Might Influence Cancer Pathogenesis). Various studies have unveiled mechanisms through which the mycobiome contributes to cancer development, either directly by acting on cancer cells or indirectly through interactions with bacteria or the immune system, subsequently affecting immune responses.

Here we discuss recent advancements in understanding the relationship between the mycobiome and cancer, as well as the potential therapeutic applications of mycobiome modulation in anticancer treatments.

## FUNGAL SIGNATURE IN CANCERS

Different types of cancer show unique mycobiomes (Table 1) (28). However, the idea that each cancer harbors a specific tumor-associated mycobiome has faced many challenges, mostly related to the lack of standardized protocols and potential risk of possible contamination during sampling and processing (29, 30). Many discrepancies in mycobiome studies were also attributed to the different types of specimens used, different laboratory techniques to isolate and characterize the fungal communities, different pathologic subtypes and stages of cancer, high inter-and intraindividual variability of the mycobiome, and, overall, the lack of common evaluation criteria. The method used for fungi identification has been a very important point of discussion over the years, including the absence of highly conserved regions such as 16S for bacteria. Sequencing the internal transcribed spacer (ITS) region of the nuclear rRNA is the gold standard in Sanger sequencing–based species detection and ITS-based methods (31, 32). However, due to its length, most of the studies focus on either the ITS1 or the ITS2 region, and the choice between the one and the other can already introduce a bias. Indeed, although focusing on the ITS2 region may reduce the taxonomic errors compared with focusing on ITS1, there are studies that reported conflicting results when comparing the fungal profiles based on ITS1 or ITS2 sequencing (33, 34). Despite these limitations, the existence of a cancer-associated mycobiome has been confirmed by several studies independently of whether they sequenced the ITS1 or the ITS2 region with the use of appropriate control tissues to rule out any contamination artifact. A comprehensive study (12) analyzed the cancer mycobiome in 17,401 samples of tumor tissue, blood, and plasma from four cohorts with 35 types of cancer, including bone, breast, colon, brain, lung, skin (melanoma), ovarian, and pancreatic. The investigation used ITS sequencing and The Cancer Genome Atlas (TCGA) whole-genome sequences for analysis (12). The study revealed the presence of fungi within tumors, often found near cancer cells and macrophages, and identified 31 fungal species with at least 1% coverage of their genomes. This group included *S. cerevisiae* (99.7% coverage), *Malassezia restricta* (98.6% coverage), *Candida albicans* (84.1% coverage), *Malassezia globosa* (40.5% coverage), and *Blastomyces gilchristii* (35.0% coverage). The study also identified low-abundance mycobiomes specific to each cancer type (12).

A separate study (27) carried out a comprehensive analysis across different body sites, recognizing diverse tumor-specific mycobiomes. The authors investigated various types of cancer from the TCGA cohort and outlined the community profiles of tumor-associated fungi with minimum genus-level resolution. In line with the previous study, they also found fewer fungal sequences than bacterial ones in tumors. They found more fungal DNA in tumor tissues from the head and neck, colon and rectum, and stomach than from the esophagus, with a negligible amount in brain tissue.

The average proportions of bacteria and fungi in TCGA primary tumors were 96% and 4%, respectively, further highlighting the lower occurrence of fungi. There was a higher prevalence of *Blastomyces* in lung tumor tissues, while *Candida* was predominant in stomach cancers. The study (27) also noted that the cancers of the gastrointestinal tract can be divided into *Candida*- and *Saccharomyces*-associated tumors based on their relative abundance. Tumors dominated by either *Candida* (Ca-type) or *Saccharomyces* (Sa-type) influenced gene expression within the tumor. Ca-type tumors showed increased expression of interleukin (IL)-1 proinflammatory immune pathways and a higher number of neutrophils, indicating a type 17 signature and suggesting a potential correlation between the mycobiome, inflammation, and cancer progression (27).

While intratumoral fungal and bacterial communities coexisted without competitive interactions (see the sidebar titled Fungi–Bacteria Interaction), they were associated with different immune responses. Three fungal-bacterial-immune clusters were identified: F1 (*Malassezia*– *Ramularia*–*Trichosporon*), F2 (*Aspergillus–Candida*), and F3 (several genera, including *Yarrowia*), each exhibiting unique interactions with their respective mycobiomes, bacteriomes, and immunomes. To define the fungal-bacterial-immune interaction, six distinct immune subtypes previously identified as C1–C6 in TCGA patients and/or patient survival (35) were used, and it was found that F1, F2, and F3 formed separate clusters, each leading to a different host response (12). The immune response was associated with higher inflammatory, lymphocyte-depleted, and strong macrophage response; however, inflammatory responses have the best survival prognosis as also reported using the same TCGA patients’ database (35). Unsupervised analyses and machine learning evaluations indicated a significant correlation between the proportions of fungus-driven mycotypes and immune response with overall patient survival across 20 different types of cancer. The results of these studies suggested the existence of a mycobiome signature in tissue and blood samples across 35 types of cancer and a positive correlation between fungal and bacterial diversity and the distinct host immune response.

However, a cause-effect relationship between these clusters and the predisposition to cancer has not been established (12). A recent metagenomic analysis on lung adenocarcinoma and the corresponding healthy tissues from patients showed that *Aspergillus sydowii* promotes the progression of lung adenocarcinoma by recruiting and activating myeloid-derived suppressor cells through IL-1β signaling, which is mediated by the β-glucan-triggered Dectin-1/CARD9 (caspase recruitment domain-containing protein 9) pathway (36). In light of these findings, future research is needed to determine whether the tumor-associated mycobiome plays a crucial role in tumor formation.

## THE MYCOBIOME AND COLORECTAL CANCER

The human colorectum harbors a dense population of microbes (20, 37–39) comprising 99% of bacteria and just 1% of fungi (40). Lifestyle factors associated with an increased risk of colorectal cancer (CRC), such as Western and low-fiber diets, excessive alcohol consumption, a sedentary lifestyle, smoking, and metabolic diseases, can influence the composition of the gut microbiome (41, 42). Although much of the research on the gut microbiome has centered on changes in its bacterial community, there is evidence to suggest that CRC is also associated with an imbalance in its fungal population (20, 43–45).

**Figure 2:**
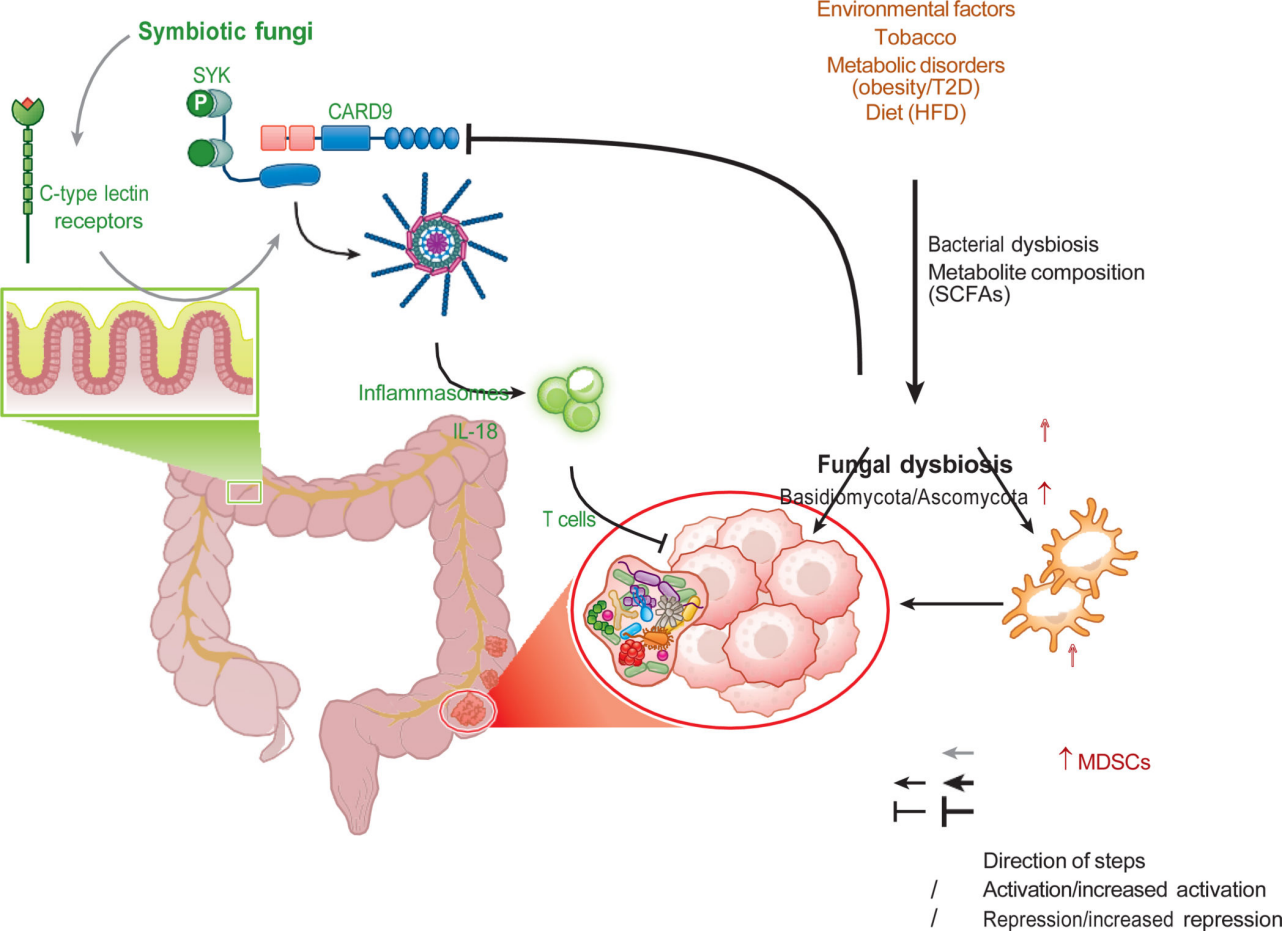
The gut mycobiome and CRC. Mycobiome signatures reveal that environmental factors such as smoking, HFD, and metabolic disorders create dysbiosis and modulate the mycobiome in association with CRC. The findings corroborate the notion of considering the gut mycobiome as a unique factor that can affect activations of inflammasomes via the SYK-CARD9 axis. Immunosuppression created by bacterial dysbiosis and altered metabolism influences fungal colonization and functions. Fungal interaction with gut bacteria will facilitate a better understanding of the CRC and microbiome modulators. Abbreviations: CARD9, caspase recruitment domain-containing protein 9; CRC, colorectal cancer; HFD, high-fat diet; IL, interleukin; MDSC, myeloid-derived suppressor cell; SCFA, short-chain fatty acid; SYK, spleen-associated tyrosine kinase; T2D, type 2 diabetes.

Studies have shown that specific bacteria and fungi in the gastrointestinal tract correlate with CRC development; specifically, fungal imbalances may lead to an increase in specific fungi potentially involved in the onset of CRC (20, 43, 44, 46). For example, the phyla Basidiomycota, Glomeromycota, and Ascomycota are abundant in CRC patients (20). An increased ratio of Basidiomycota to Ascomycota is associated with a higher potential for pathogens such as *Rhodotorula*, *Malassezia*, *Acremonium*, and *Aspergillus* spp., which are known to carry carcinogen-producing genes (Figure 2) (20). Furthermore, fecal shotgun metagenomic sequencing of patients with CRC suggests that fungi in the gastrointestinal tract play a role in CRC progression (20, 25, 37, 46).

In a study involving 131 subjects, which included both CRC patients and healthy individuals, an increased abundance of opportunistic fungi such as *Trichosporon* and *Malassezia* was observed in CRC patients as compared with healthy individuals. Moreover, an increased ratio of Basidiomycota/Ascomycota was proposed as a marker of fungal dysbiosis in CRC (44). This ratio notably increased during CRC progression (20).

The same research suggested that gut mycobiomes could predict early- and late-stage CRC. Analyses of fungal species in CRC also led to the identification of 14 fungal species that were differentially expressed between the CRC group and the controls (20, 37). Interestingly, *Lipomyces starkeyi* and *S. cerevisiae,* which may contribute to dysbiosis due to their known potential to inhibit pathogen growth and alleviate inflammation, were less abundant in CRC individuals (20).

Another large-scale investigation involving 1,368 samples from eight different geographic cohorts revealed a correlation between specific fungal and bacterial species, such as *Talaromyces islandicus* and *Clostridium saccharobutylicum*. This study also suggested that elevated d-amino acids and butanoate metabolism could serve as potential CRC biomarkers (45).

In a recent study, a higher abundance of the *Candida* fungus was linked to stomach cancers and increased proinflammatory immune pathways, further suggesting that the gut mycobiome plays a role in tumor growth and inflammation (27). In the case of CRC, *Candida*’s presence may indicate either late-stage disease and metastases or a weakening of cell adhesion mechanisms that would result in the disruption of the intestinal barrier (27).

It is essential to note, however, that while cancer-associated imbalances in gut microbes may be a cause of colorectal inflammation and subsequent CRC onset, a healthy gut relies on commensal fungi and bacteria for intestinal homeostasis (27, 45). Fungal dysbiosis, occurring under pathological conditions, may lead to the elimination of some bacterial species, impacting corresponding immune responses and potentially increasing disease susceptibility, as observed in nonalcoholic fatty liver disease (47, 48).

Fungal dysbiosis is often associated with CRC risk factors such as obesity and high-fat, low-fiber diets lacking necessary micronutrients such as vitamins and minerals (49, 50). High-fat diets may alter the gut mycobiome and impact immune-metabolic networks crucial in CRC development (51). This microbial imbalance may also catalyze colorectal inflammation, thus contributing to CRC pathogenesis (27, 52).

In a clinical study, high *Candida* levels were associated with increased IL-1 expression, an inflammatory cytokine involved in inflammation and tumor progression (27, 53–55). The interplay between harmful and symbiotic gut fungi could also influence CRC pathogenesis. CARD9 expression increases in CRC biopsies and immune cells during tumorigenesis (56, 57). Under normal conditions, symbiotic fungi are recognized by C-type lectin receptors, triggering downstream signaling via spleen-associated tyrosine kinase (SYK) and CARD9 (56).

In CRC mouse models, the activation of inflammasomes supports antitumor T cell immunity by promoting IL-18 maturation. The protective T cell response induced by symbiotic fungi may limit CRC progression (56). However, mycobiome dysbiosis can inhibit the antitumor SYK-CARD9 axis and its protective immune response, thereby promoting CRC (56).

Replication of *Candida tropicalis* in macrophages directly affects the population of myeloid-derived suppressor cells (MDSCs)—a diverse group of innate immune cells that have been suggested to inhibit cytotoxic T cell responses—potentially leading to CRC (57). Conversely, CARD9 signaling activated by symbiotic fungi may decrease the number of MDSCs in the colon, potentially mitigating CRC pathogenesis and tumor growth in mice (57).

The mycobiome could potentially influence the development of CRC by controlling multiple events such as the regulation of inflammation, immune response, and progression from adenoma to carcinoma (58). Recent research has highlighted the role of intestinal metabolites in CRC development (45). Both bacterial and fungal metabolism generate short-chain fatty acids, such as acetate, propionate, and butyrate, which are implicated in CRC’s development (59). In particular, acetate amplifies the presence of type 3 innate lymphoid cells (ILC3s) in the colon, and propionate increases the number of IL-22+ ILC3s. These shifts might eventually decrease inflammation in the colon and reduce the probability of CRC.

In cases of CRC, the presence of *Candida* predicts advanced stages of the disease and metastasis and correlates with weakened cellular adhesion functions. A comparison of fungal and bacterial communities inside tumors showed coexisting yet noncompetitive environments, with separate immune responses (12, 27, 45). Thus, fungal imbalance could promote cancer by disturbing healthy immune responses to gut bacteria, suppressing anti-inflammatory processes stimulated by commensal fungi, or by encouraging the buildup of cancer-causing toxins.

Considering that CRC accounts for 10% of global cancer incidence and 9.4% of cancer-related deaths, there is an immediate need to improve our understanding of the mycobiome’s role in CRC while focusing on its involvement in CRC development.

## THE MYCOBIOME AND HEAD AND NECK CANCER

**Figure 3:**
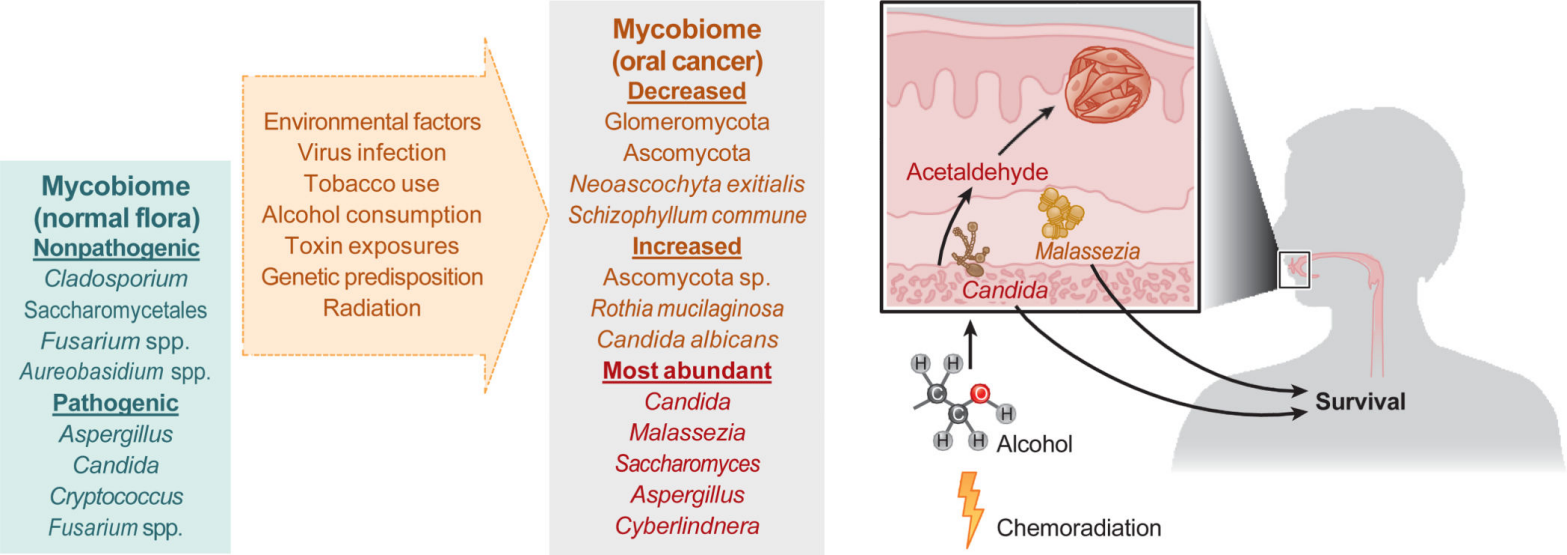
The oral mycobiome and head and neck cancer. *Candida* and *Malassezia* spp. are the most commonly encountered fungi in the oral mycobiome of head and neck squamous cell carcinoma. Factors that affect the oral mycobiome include viral infection, tobacco use, radiation, and genetic predisposition. Fungi, specifically *Candida albicans*, can metabolize alcohol to the carcinogen acetaldehyde.

Oral cancer (OC) and oropharyngeal carcinoma are the most frequent metastatic head and neck cancers in humans (60). The oral cavity acts as a natural reservoir for both harmless and opportunistic fungal species that can colonize various habitats particularly in the head and neck regions (Figure 3) and include nonharmful fungi such as *Cladosporium*, Saccharomycetales, *Fusarium* spp., and *Aureobasidium* species and harmful fungi such as *Aspergillus*, *Candida*, *Cryptococcus*, and *Fusarium* spp. (19, 23).

In adults, *Candida* species, especially *C. albicans*, are commonly found in the oral cavity. An excessive presence of these opportunistic pathogens can contribute to disease in the head and neck by interacting with acid-producing bacteria and promoting biofilm production (61–63).

A study of leukemia patients’ oral mycobiomes revealed fungal imbalances leading to invasive mucormycosis (64). The Glomeromycota and Ascomycota species were less prevalent in tongue cancer patients compared with healthy individuals (65–69).

Also, numerous bacterial and fungal communities (e.g., Ascomycota species, *C. albicans*, and *Rothia mucilaginosa*) were abundant in head and neck squamous cell carcinoma (HNSCC) patients but were absent in healthy individuals (70). *Neoascochyta exitialis* and *Schizophyllum commune* were underrepresented in the oral cavities of HNSCC patients (65, 66, 70). Interestingly, the prevalence of certain *Candida* and *Aspergillus* species was linked to cancer incidence (71–73).

Notably, higher levels of *C. albicans* were strongly associated with dysbiotic mycobiomes in oral squamous cell carcinoma (OSCC) cases (63). One study identified 16 unusual fungal genera in the saliva of OSCC patients, with *Candida*, *Malassezia*, *Saccharomyces*, *Aspergillus*, and *Cyberlindnera* being among the most prevalent (74). In OSCC patients, the presence of *Candida* was linked to worse survival rates, while the presence of *Malassezia* correlated with improved survival rates (74). In summary, a notable shift in *Candida* species appears to occur during the development of OSCC.

Key factors that contribute to HNSCC include viral infections such as human papillomavirus, habitual tobacco and alcohol use, genetic predisposition, repeated exposure to ultraviolet (UV) radiation, and contact with carcinogens such as asbestos and formaldehyde (60). The composition of the oral microbiome is impacted by smoking and alcohol use (75–78). These chemicals further interact with oral bacteria and fungi, resulting in harmful by-products.

Increased acetaldehyde production by microbes, associated with alcohol consumption, has been linked to OC (79). Fungi may contribute to cancer development directly through carcinogenic metabolites or indirectly by disrupting epithelial membranes, thereby promoting inflammatory responses and microbiome imbalance (80). The metabolism of the fungus *Candida* may play a role in cancerous changes through the generation of nitrosamines (81) and acetaldehyde (68–70, 79).

Heightened levels of Dectin-1, a critical receptor for antifungal immunity, were observed in cases with a higher presence of *Candida albicans*. An increase in Dectin-1 signaling can shift the immune environment and foster tumor growth (82). Additionally, *C. albicans* can cause an increase in oncogenes, contributing to all stages of OC—from early development to metastases. This is achieved by stimulating the production of matrix metalloproteinases, onco-metabolites, and protumorigenic signaling pathways and by the overexpression of genes that initiate metastatic events (83).

## THE MYCOBIOME AND PANCREATIC CANCER

**Figure 4:**
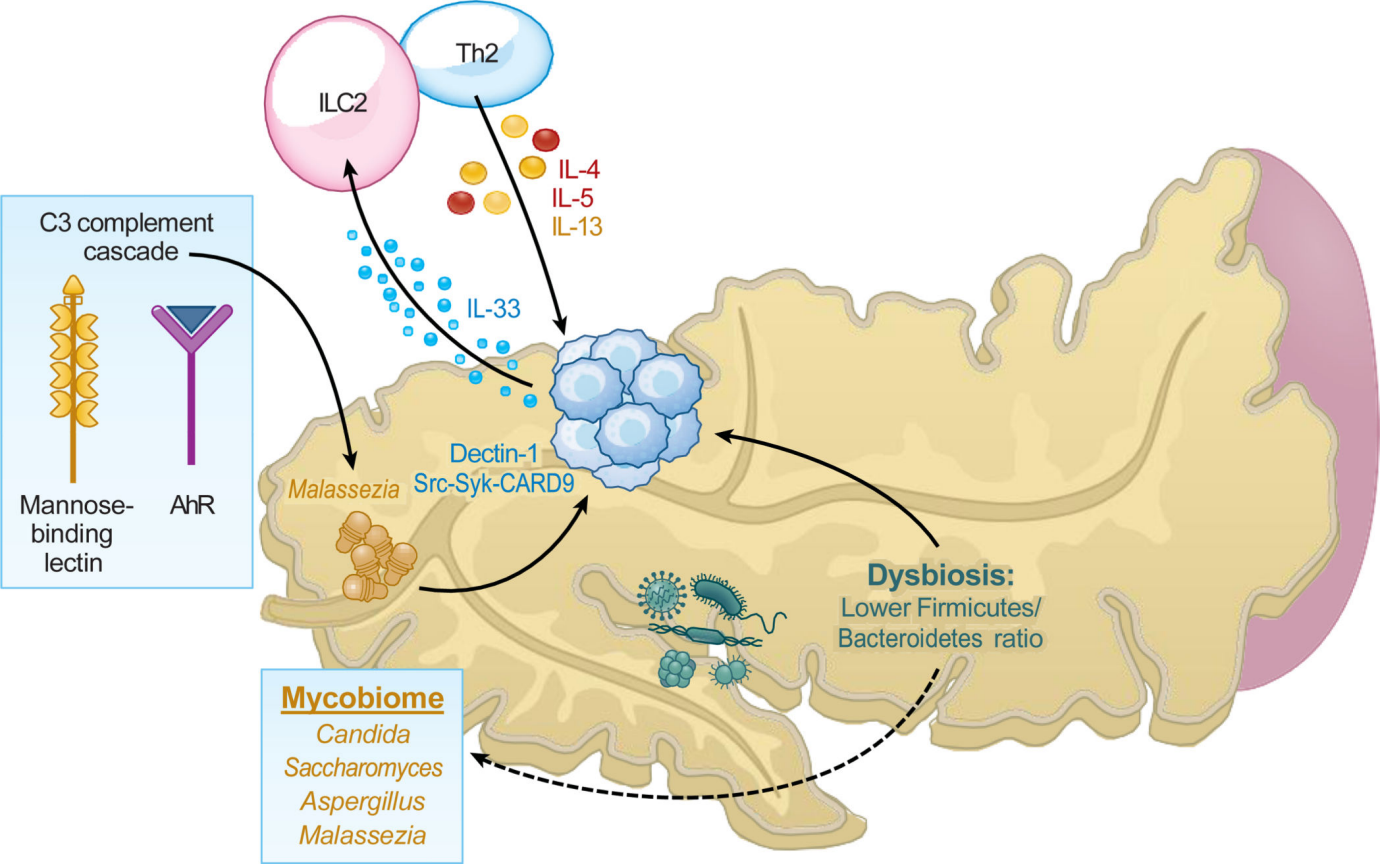
The TME mycobiome regulates PDAC immunity. Members of the mycobiome activate the complement cascade and promote tumor progression in PDAC. The intratumoral mycobiome releases IL-33, which promotes Th2 and ILC2 cell infiltration and increases the PDAC burden. Bacteria in the TME can have immunosuppressive activity and interact with fungi to modulate the host immune response and induce tumor progression. The dashed arrow indicates an unconfirmed pathway. Abbreviations: AhR, aryl hydrocarbon receptor; CARD9, caspase recruitment domain-containing protein 9; IL, interleukin; ILC2, type 2 innate lymphoid cell; MBL, mannose-binding lectin; PDAC, pancreatic ductal adenocarcinoma; Syk, spleen-associated tyrosine kinase; Th2, T helper 2; TME, tumor microenvironment.

Earlier studies suggested that the pancreas was a pathogen-free organ (84). However, recent evidence indicates that the pancreas, in fact, houses various microbial populations, including bacteria and fungi. These microbes potentially play key roles in the development and progression of pancreatic ductal adenocarcinoma (PDAC) (85) (Figure 4). There are four primary ways that microbes could colonize the pancreas. The first pathway involves microorganisms from the oral cavity, stomach, intestine, or biliary duct entering the pancreatic tissue via the pancreatic duct. Next, microbes may reach the pancreas from the intestine via the lymphatic system. Alternatively, bacterial translocation to the pancreas may occur through the mesenteric vein that drains into this organ (86, 87). Lastly, microbes can cross the gut’s vascular barrier, enter the bloodstream, and reach the pancreas through the systemic circulation (88). For instance, gut mycobiomes can colonize the pancreas via the sphincter of Oddi (22). Independent of the entry route, it is currently hypothesized that the pancreatic mycobiome significantly impacts pancreatic functions and diseases (12, 21, 22, 27).

Our recent study identified Ascomycota and Basidiomycota as the most common phyla in both human and mouse gut and pancreatic tumors (22). In agreement with our findings in mice, *Malassezia*, a specific genus, was more prevalent in human PDAC tissues than in the gut. We further demonstrated that oral gavage with *M. globosa* in mice results in fungal translocation from the gut to the pancreas while also accelerating PDAC progression in a complement-dependent manner. Interestingly, our findings showed that the mannose-binding lectin (MBL) that interacts with the glycans in the fungal wall activated the complement cascade via the lectin pathway, ultimately contributing to carcinogenesis. Pancreatic cells lacking MBL or C3 resisted carcinogenesis through the MBL pathway (22), confirming the role of MBL-induced complement activation in PDAC development. Molecular studies further confirmed MBL’s involvement in PDAC pathogenesis (22). Further, transcriptomic data from TCGA indicated a correlation between higher *MBL2* expression in PDAC patients and reduced survival.

We identified a ∼3,000-fold increase of *Malassezia* in the PDAC of both mice and humans as compared with controls. We showed that an antifungal drug, amphotericin B, significantly slowed down the progression of invasive PDAC tumors (22). Conversely, reintroducing *Malassezia* into mice triggered carcinogenesis, an effect not seen with other commensal fungi such as *Candida*, *Saccharomyces*, or *Aspergillus* in mice (22).

Further evidence for the role of the mycobiome in PDAC pathogenesis came from a recent mouse study (21) showing that PDAC tumors are infiltrated by *Alternaria* and *Malassezia*. The study used a mouse model to demonstrate that the intratumoral mycobiome activated Dectin-1-mediated Src-Syk-CARD9 signaling in the pancreas. This led to the release of IL-33 (21), a key cytokine for T helper 2 (Th2) and ILC2 cells. The mycobiome spurred the discharge of IL-33, which then propelled tumor growth. This process was facilitated by the release of protumorigenic cytokines, including IL-4, IL-5, and IL-13 (21). Notably, restricting IL-33 and eradicating the pancreatic intratumoral mycobiome using antifungal treatments significantly reduced the infiltration by Th2 and ILC2 cells and increased the survival rates of the tumor-bearing mice (21). In addition, *Malassezia*’s secretome could partake in indirect effects on PDAC progression. These yeasts produce potent indolic ligands that target the aryl hydrocarbon receptor (AhR), a transcription factor that plays important roles in the UV-damage and host immune responses (89). Notably, increased AhR expression correlates with gastric cancer progression and metastases (90) and with pancreatic cancer (91).

Following the publication of our results, Fletcher et al. (92) reanalyzed our data using newly updated DADA2 and QIIME2 analysis tools as bioinformatic platforms and showed that their analysis identified fewer fungal reads in the PDAC tissues. Although we used QIIME1 and not DADA2 for the analysis of the published data, when conducting the reanalysis with QIIME2, we still confirmed our previous findings (93) and identified 2,980 operational taxonomic units (OTUs) in the feces and 311 OTUs in the PDAC tissues after removing low-abundance OTUs. The discrepancy between the two analyses pipelines may very likely be dependent on the different tools utilized to demultiplex the sequences. Moreover, the results can be influenced by the various analytical techniques used, such as the type and amount of tissue sample extraction, DNA isolation (fungal DNA), sequencing, bioinformatic pipelines, and reference databases (12). Our conclusions for a role of fungi in PDAC are based not solely on sequencing data but also on widely used animal models of PDAC that allowed us to perform in-depth mechanistic studies (22, 93). Furthermore, several other foundational and translational studies, including a recent publication from the PDAC Biomarker Working Group, corroborated the significance of *Malassezia* and other fungi in PDAC (12, 21, 94, 95). Recently, Galloway-Pena et al. (96), in a viewpoint article, suggested that *Malassezia* may be a contaminant by using a protocol to remove potential contaminant from analysis (97), whereas Narunsky-Haziza et al. (12) used their own analysis protocol and showed the presence of *Malassezia* in many cancers. We used all the quality control steps including negative controls and mock community in our analysis and found *Malassezia* to be a prominent fungi associated with PDAC (22, 93). Currently, there is no clear consensus among the microbiome community about how to deal with low-biomass and low-abundance sequences, as even these low-abundance fungi may have biologic relevance in the context of cancer. It is worth noting that identification of *Malassezia* in many cancers including breast, colorectal, pancreatic, and oral cancers raises an intriguing question about *Malassezia* in cancer pathogenesis and cannot be ruled out as a mere coincidence or contamination (12, 21, 22, 74, 94, 97–99). Large-scale investigations will be needed in the future to delineate the exact role of the mycobiome in PDAC, and a role for fungi in pancreatic oncogenesis cannot be dismissed (93).

PDAC accounts for roughly 3% of all cancer cases in the United States and approximately 7% of all cancer-related deaths (60, 100, 101), and its late diagnosis often renders surgery ineffective. Therefore, understanding the potential involvement of the gut mycobiome in the pathogenesis of PDAC (Figure 4) suggests the possibility of exploiting the mycobiome to identify not only more effective treatments but also preventative strategies to diagnose the disease at earlier time points when treatments may result in more positive outcomes.

## THE MYCOBIOME AND THE COMPLEMENT SYSTEM

The mycobiome plays a key role in health and disease development. Illnesses can alter not only the bacterial components of the gut microbiome but also the mycobiome’s composition. For example, colon cancer patients, even at the polyp stage, may exhibit a higher Ascomycota/Basidiomycota ratio and an increased proportion of opportunistic fungi such as *Trichosporon* and *Malassezia* (44). It is intriguing to note that obese individuals and those with inflammatory bowel disease often show increased levels of *Candida* spp., suggesting its potentially harmful effects under various pathological conditions (102, 103).

The fungal species dwelling in gastrointestinal tracts are vital in the immunological aspect of gut microbiota. They interact symbiotically with the host in stable conditions. A functional immune system in a healthy person acts as a guard against fungal overgrowth. *Candida albicans*, for instance, is a common pathogenic fungus in immunocompromised individuals, causing local and systemic life-threatening infections (104).

The complement system provides the first line of defense against fungal pathogens. Studies have shown that mice deprived of key complement proteins such as C3 or C5 are more susceptible to *Candida* infections (105, 106). Furthermore, a hereditary deficiency of C5 or treatment with the US Food and Drug Administration–approved anti-C5 monoclonal antibody (eculizumab) often leads to severe, life-threatening fungal infections, highlighting the complement system’s essential role in tackling fungal pathogens (107, 108). This higher susceptibility to infection is dependent on the complement anaphylatoxin C5a and its receptor, C5aR, which play a key role in attracting and licensing macrophages to kill pathogenic fungi via nonoxidative mechanisms (109). Although *Candida* can activate all three pathways of the complement system, growing evidence suggests that the MBL pathway plays a central role in activating *Candida* phagocytosis by neutrophils (110, 111).

Interestingly, fungal species have developed evasion mechanisms to avoid complement activation (112, 113). Notably, *Candida* can resist complement activation by retaining complement regulators from the plasma on its surface or blocking the activation using proteins such as Pra1, Sap1, Sap2, and Sap3. These factors prevent complement activation by directly binding C3 or by degrading downstream activation molecules such as C3b, C4b, and C5, ultimately blocking downstream complement effector mechanisms necessary for fungal elimination (114). Pathogenic fungi such as *Aspergillus*, *Histoplasma*, and *Blastomyces* also employ such strategies to persist and evade antifungal actions by the complement system (115).

In cancer patients, the interaction of fungal species and the complement system can yield contrasting outcomes. As our findings show, an imbalanced gut biome may trigger the growth of pathogenic fungi such as *Malassezia*, which promotes tumor growth by activating the complement system (22). However, in patients undergoing radiotherapy or chemotherapy, a weakened immune system may catalyze the proliferation of fungal species that inhibit complement activation, leading to life-threatening systemic infections.

## TARGETING THE MYCOBIOME FOR CANCER THERAPY

Recent advancements in the comprehension of the mycobiome and its implications in cancer pathogenesis suggest potential alternatives for more effective cancer prevention and treatment. Intriguingly, a study by Shiao et al. (116) indicates that the intestinal mycobiome manipulates antitumor immune responses postradiation in mouse models of breast cancer and melanoma. The study shows that fungi and bacteria inversely affect these responses. Specifically, radiation therapy’s responsiveness is improved by treating with antifungal drugs, whereas antibiotic treatment causes a decrease in the radiation therapy response due to the proliferation of commensal fungal species.

These findings hold importance for cancer patients, since the authors discovered that a higher intratumoral expression of Dectin-1 correlated negatively with breast cancer survival rates. Dectin-1 was found to be necessary to mediate the fungal inhibition of radiotherapy (116). A related study by Bukavina et al. (117) in bladder cancer patients revealed that chemotherapy responders showed a higher prevalence of Hypocreales and a lower prevalence of Saccharomycetales than nonresponders. Conversely, nonresponders had an increased abundance of Agaricomycetes and Saccharomycetes. With these results, the authors hypothesize that a “favorable” mycobiome composition may boost the systemic immune response to chemotherapy via antigen presentation (117).

In summary, these studies propose the possibility of treating gut dysbiosis with the goal of restoring the gut’s healthy microbiome. This could be achieved by eradicating intratumoral pathogenic fungi using antifungal agents (Figure 1).

## TREATING FUNGAL DYSBIOSIS BY MODULATING DIET

Besides genetic and environmental factors, fungal dysbiosis can predispose patients to pancreatic cancer and CRC. The progression of these cancers may be altered by reversing fungal dysbiosis through dietary modifications and probiotic use. Diet plays a crucial role in determining the gut mycobiome and bacteriome compositions (118). The abundance of *Candida* and *Saccharomyces*, connected with gastrointestinal cancers, is influenced by the diet. An increased presence of *Saccharomyces* in the gut is linked to meat consumption (119). Similarly, a high-intake diet rich in hemicellulose arabinoxylan and low in total saturated fatty acids correlates with gut colonization by *Candida* (118). Additionally, the presence of *Aspergillus* spp., which is a potent aflatoxin producer in the gut, is associated with hepatocellular carcinoma (38) and negatively correlates with the intake of short-chain fatty acids (118).

An in vitro study suggests that β-glucan, extracted from *S. cerevisiae*, could protect against DNA damage, implying potential benefits as a dietary supplement. Ideally, adjusting one’s diet to alter the gut mycobiome and counteract the dysbiosis related to cancer would be a noninvasive strategy. However, we need more insight into the functions and signatures of the cancer mycobiome to offer individual guidance and encourage a sustained dietary commitment to prevent cancer and hinder tumor progression.

## PROBIOTICS FOR TREATING DYSBIOSIS

Prebiotics and probiotics can potentially modify fungal diversity (119–129). *Saccharomyces cerevisiae* serves a crucial protective role against cell damage and genotoxicity. Glucan, extracted from fungi such as *S. cerevisiae*, may act as a prebiotic, protecting against DNA damage cytotoxicity and potentially preventing cancer (123). A probiotic blend containing beneficial fungi from the *Saccharomyces* genus, along with bacteria from the *Lactobacillus* and *Bifidobacterium* genera, displays anti-CRC properties (120). Similarly, *S. boulardii* reduces gut inflammation, thereby inhibiting colitis-related cancer (125). This effect is due to its ability to produce high concentrations of acetic acid in the gut (128). In a mouse model of *Clostridium difficile* infection, *S. boulardii* suppressed inflammation and tumor development by inhibiting certain cell signaling pathways, leading to increased tumor cell death (124).

Apart from diet adjustments and probiotic intake, fecal microbiota transplantation (FMT), including bacteria and fungi, can lessen fungal imbalance (130–132). FMT is often applied as a complementary therapy in immunotherapy for specific epithelial cancers such as CRC (133). Interestingly, FMT with *C. albicans* tends to limit the efficacy of the procedure. However, research on a mouse model with *Clostridium difficile* infection indicated that the effectiveness of FMT correlated with the colonization of *Saccharomyces* and *Aspergillus* but not *Candida* (131). This suggests that some fungal species may significantly impact the therapeutic success of FMT. Despite these findings, most studies emphasize bacterial colonization after FMT. Thus, our comprehension of the mycobiome’s role in FMT remains limited. Future research is needed to understand the direct or indirect role of the mycobiome in affecting the efficacy of FMT.

## ANTIFUNGAL THERAPY AND ITS LIMITATIONS

Antifungal chemoprophylaxis is vital in clinical practice, given the heightened risk for life-threatening fungal infections in cancer patients undergoing chemotherapy or receiving allogeneic stem cell transplants for hematological cancers (134). However, our understanding of the role of antifungals as adjuncts in cancer treatment remains limited. Two recent studies emphasized the value of antifungals in PDAC therapy. Using amphotericin B or fluconazole in treating tumor-bearing mice significantly hindered the progression of PDAC (21, 22). Furthermore, eradicating the mycobiome with antifungals decreased PDAC tumor load and boosted the efficacy of the chemotherapy drug gemcitabine, hinting at a possible combination therapy for pancreatic cancer (22, 135).

Itraconazole, routinely used in managing various fungal infections such as candidiasis, as-pergillosis, blastomycosis, and histoplasmosis, can also limit tumor angiogenesis (136, 137). There are suggestions that itraconazole could help slow down the advancement of various cancers, such as prostate, lung, ovarian, breast, and pancreatic cancers, as well as leukemia (136, 138); however, more clinical data are required for such cancer treatments.

While the results appear promising, the use of antifungal drugs still has significant constraints. Most clinical trials involving antifungal drugs, such as fluconazole and itraconazole, have been conducted prophylactically. These drugs, available in oral form and approved for antifungal prevention, work by inhibiting ergosterol synthesis, the key fungal sterol (139). However, studies show that these drugs often interact with other medications, particularly chemotherapy drugs, posing a significant obstacle to their use in cancer patients (140).

Another class of antifungal agents, echinocandins, which specifically inhibit the fungal 3β-glucan synthase, show linear pharmacokinetics over a broad dosage range. They have a long half-life, reducing the need for repeated dosing, and are generally well tolerated. Unlike triazoles, echinocandins do not significantly interact with other drugs (141). However, despite their fungicidal activity against most *Candida* species, in vitro studies reveal that they possess cytostatic activity only against most *Aspergillus* species (142).

Amphotericin B, an antifungal from the polyenes family, works by interacting with the sterols on a fungal cell membrane. This interaction leads to the creation of pores, altering membrane permeability, causing leakage of cellular components, and, ultimately, inducing cell death (142). This drug has the most extensive fungicidal activity range. However, its broad scope of toxicity has been partially addressed with innovative liposomal or lipid complex formations (140).

One of the drawbacks of long-term use of antifungal agents is the emergence of dysbiosis that affects not only fungal but also bacterial communities of the microbiota, subsequently impacting local and peripheral immune responses. For example, animal studies illustrate that treatments with drugs such as fluconazole or amphotericin B can exacerbate chemically induced or T cell–driven intestinal inflammation and increase airway inflammation from house dust mites.

It was found that, in addition to promoting the growth of resistant fungal species, these anti-fungal treatments also restructured bacterial communities, resulting in a substantial decrease in some protective *Lactobacillus* species (143). Given that bacteria and fungi in the intestine occupy similar niches, it is plausible that they can influence each other.

Considering these findings, additional research should focus on assessing how fungal and bacterial species within the human body interact to modulate immune responses. Also, future studies should consider exploring promising compounds and conducting rigorous pharmacokinetic/ pharmacodynamic analyses, particularly regarding extended dosing intervals. This is vital for future cancer patients’ combination therapy.

## CONCLUSIONS AND FUTURE DIRECTIONS

### Toward a Better Understanding of the Relation Between Fungi and Cancer

Next-generation sequencing techniques, bioinformatics, and comprehensive data analysis have uncovered potential links between the mycobiome and human diseases, including cancer. Fungi are part of the normal microbiota in various organs and tissues. As such, their disruption may lead to diseases. Research has revealed the presence of the mycobiome in many cancers, especially those related to the digestive system, such as CRC, PDAC, and HNSCC. However, most of the studies are association studies, and very few studies have suggested causation (144). We observed a shift in the mycobiome in a predisposed genetic mice model, suggesting disease progression; however, it is not clear if fungi can cause cancer. There is a possibility that genetic predisposition or environmental factors play a significant role in altering the mycobiome, which in turn can accelerate disease progression.

Still, mycobiome research is in its early stages, mainly hampered by the absence of universal sequencing targets applicable to all fungi. This challenge is key to understanding full fungal diversity. Additional needs include specific reagents such as polymerase chain reaction primers for different fungal types, special databases for fungal bioinformatics analyses, and more funding dedicated to mycobiome research. Without these resources, it will be difficult to map the mycobiome diversity, uncover new fungal species, and understand their relationships with their hosts and other microbiome components.

Further studies should examine the complex relationship between gut-dwelling fungal en-dosymbionts, their produced metabolites, and the influence of these elements on other organs (45). Multiple tumor types display distinct fungal signatures (12, 21, 22, 25–28), suggesting potential implications for cancer therapy (145, 146). For instance, dysbiosis could be treated with antimi-crobial therapy, dietary modification, or either of these treatments combined with chemotherapy or immunotherapy.

The potential is evident in the diagnostic and prognostic capabilities of tissue and plasma mycobiomes for cancer diagnoses (145, 146). One clinical study revealed a signature of circulating fungal DNA from 20 distinct fungi, which differentiated healthy individuals from those with pan-cancer (12). This highlights the role of mycobiome and machine learning tools in cancer diagnostics, even in early-stage patients. We need more hypothesis-driven studies involving significant patient cohorts to unravel the mycobiome’s role in cancer pathogenesis (see the sidebar titled Targeting the Mycobiome May Have Clinical Implications). Evidently, the influence of the mycobiome is not limited to causing primary and secondary infectious disorders; it also potentially has substantial roles in the initiation and progression of many cancers.

### Current Limitations in Mycobiome Research

First, the human mycobiome is much smaller than the bacterial microbiome, which complicates whole-community analyses as it requires a significantly increased sequencing depth to capture the rare mycobiome (4). Moreover, the biological differences between fungal and prokaryotic organisms influence DNA extraction, purification, and subsequent analysis (147, 148). Sample size and sample type, clinical information, inference across most human cancer types, experimental contamination controls, differences in sample preparation, sequencing, bioinformatic pipelines, and reference databases are some key factors that can influence mycobiome studies. Furthermore, the relative lack of fungal data in public repositories, coupled with discrepancies in recorded environmental fungal diversity, adds complexity to the interpretation of mycobiome sequencing data and predictions of the ecological importance of fungi in comparison with bacteria (149, 150). This sparsity of data, along with the ongoing evolution of fungal taxonomy, hampers cross-study comparisons and standardization (151). Other impediments include significant variations in ribosomal RNA (including ITS regions) copy numbers that confound relative abundance estimates in primer-based studies (152), as well as difficulties in designing broad-coverage primers and identifying optimal amplicon length and target region (147, 153, 154). Given these difficulties, it is imperative to develop more resources and standard protocols for sequencing and mechanistic studies to understand the influence of the mycobiome on cancer development.

## Figures and Tables

**Table 1:** Association of mycobiome and various cancers

Cancer type	Fungi	Mechanism	Reference(s)
Pancreatic	*Malassezia* *Alternaria* Saccharomycetes Dothideomycetes Malasseziomycetes	Immune suppression Mannose-binding lectin (MBL)-C3 axis activation Pancreatic intratumoral mycobiome modulates the immune system to express interleukin (IL)-33, which releases protumorigenic cytokines and increases tumor growth in a mouse model	12, 21, 22
Oral	*Candida albicans* Glomeromycota Ascomycota in lower relative abundances	In cases of chronic alcohol consumption, *C. albicans* could produce acetaldehyde, which is linked to the incidence of oral cancer Dysbiosis may lead to the expansion of pathogenic fungi compared with the nonpathogenic	19, 67, 68, 70
Colorectal (includes colon and colon metastatic)	Saccharomycetes Malasseziomycetes Increase in the ratio of Basidiomycota/Ascomycota	Not known Change in metabolite composition that affects gut immune-metabolic networks in a way that promotes pathogenesis in colorectal cancer	20, 25, 27, 37, 44, 46, 56
Breast	Saccharomycetes Dothideomycetes Sordariomycetes Malasseziomycetes	Not known	12
Lung	*Malassezia* Saccharomycetes *Candida* Dothideomycetes Sordariomycetes Malasseziomycetes	Upregulation of proinflammatory cytokines, especially IL-6	12, 27
Skin (melanoma)	Saccharomycetales Saccharomycetes Malasseziomycetes Dothideomycetes	Not known	12, 27
Glioblastoma multiforme	Dothideomycetes Malasseziomycetes Microbotryomycetes Wallemiomycetes	Not known	12
Ovarian	Dothideomycetes Sordariomycetes Malasseziomycetes Agaricomycetes Tremellomycetes	Not known	12
Bone	Saccharomycetes Dothideomycetes Sordariomycetes	Not known	12

## EMERGING MECHANISMS BY WHICH THE MYCOBIOME MIGHT INFLUENCE CANCER PATHOGENESIS

Many factors contribute to fungal pathogenesis, which involves a complex interplay of many fungal and host factors.

1. Modulation of host immunity:
  - The intratumoral mycobiome found in pancreatic ductal adenocarcinoma (PDAC) activates the expression of interleukin (IL)-33, a proinflammatory cytokine chemoattractant for type 2 immune cells (21).
  - In a mouse model of PDAC, antifungal treatment markedly improves survival by decreasing tumor progression (21).
  - Fungi activate mannose-binding lectin (MBL), a protein that activates the complement cascade, which in turn promotes PDAC (20).
  - PDAC tumor progression was inhibited in mice lacking MBL, or C3, a vital constituent of the complement cascade (19).
  - In a mouse model of colorectal cancer (CRC), fungi-induced caspase recruitment domain-containing protein 9 (Card9) activation suppresses inflammation and activates T cells (56).
  - *Candida tropicalis* replication in macrophages controlled by CARD9 induces the differentiation of immunosuppressive myeloid-derived suppressor cells (MDSCs) in a mouse model of CRC (57).
2. Secretion of hydrolytic enzymes and carcinogens:
  - *C. albicans* produces specific hydrolytic enzymes able to convert alcohol to acetaldehyde (ACH), a group 1 carcinogen (155).
  - *Candida* species produce host tissue nitrosamines, such as *N*-nitrosobenzylmethylamine (NBMA) and other potential human carcinogens (35–37).
  - Aflatoxins produced by *Aspergillus* spp. are carcinogens causally linked to hepatocellular carcinoma (38).
3. Altering gene expression:
  - Elevated levels of *Candida* affect gene regulation in tumors of various cancers, including upregulation of genes that encode proinflammatory cytokines, especially IL-1 (12, 27).
  - Deregulation of genes that mediate cellular adhesion causes deterioration of the epithelial barrier, metastasis, and translocation of fungi (25).

## FUNGI–BACTERIA INTERACTION

Interactions between fungi and bacteria play a key role in the functioning of the host. Members of microbiome communities drive biochemical cycles and contribute to both health and diseases in the host.

- Components of microbiomes can interact directly by physical contact, through small molecule secretion, or upon environmental alterations such as changes in pH, the presence of particular metabolites, and host response to the mycobiome.
- Bacteria and fungi can influence each other’s growth, leading to dysbiosis and effects on colonization and attachments to epithelial cells (27).
- *Lactobacillus* spp. affect the attachment of both *Helicobacter pylori* and *Candida* spp. in stomach cancer (156).
- Fungal-driven, pan-cancer mycotypes may have distinct immune responses (12).

## TARGETING THE MYCOBIOME MAY HAVE CLINICAL IMPLICATIONS

The gut is home to a diverse mycobiome, and it is possible that these fungi can make their way to different organs. The evidence below suggests the potential of targeting the mycobiome to open up new avenues in cancer treatment.

- Tumor mycobiome diversity and composition influence pancreatic cancer outcomes (22).
- Gut fungi may migrate from the intestinal lumen to the pancreas (22).
- The gut mycobiome modulates the PDAC tumor microbiome landscape (12, 21, 27).
- Fungal wall glycans activate the complement cascade (22, 135).
- Repopulation with *Malassezia*, but not with other fungi, promotes tumor progression in mice. MBL or C3 deletion or C3aR knockdown in cancer cells is tumor protective (22).
- *Malassezia* and *Alternaria* are the most abundant fungi in PDAC. Depletion of fungi or deletion of IL-33 in cancer cells significantly decreases PDAC tumor progression and significantly decreases tumor burden (21).
- Fungus-driven, pan-cancer mycotypes with distinct immune responses can predict survival (12).
- Fungi interact with bacteria through various mechanisms and can impact one another’s survival or virulence (12, 27).
- The mycobiome is modifiable by antifungal and/or probiotics treatments, and mycobiome-targeted therapies are a promising new frontier for cancer diagnostics and treatment (12, 21, 22, 27, 127, 135, 138).

## SUMMARY POINTS

1. The human mycobiome contributes to cancer development.
2. Different cancer types each have a unique mycobiome.
3. The mycobiome can modulate host immune responses via the mannose-binding lectin (MBL) and Dectin-1 pathways.
4. Fungal species contribute to tumor induction along the gastrointestinal tract by producing carcinogens.
5. The abundance of *Malassezia* was linked to the progression of colorectal cancer, head and neck squamous cell carcinoma, and pancreatic ductal adenocarcinoma.
6. A high abundance of fungi such as *Candida* can alter gene regulation in various cancers.
7. Notwithstanding the rich diversity of the mycobiome in gut and tumor samples, the mycobiome is less diverse than the bacteriome. Mycobiome interactions with the bacteriome and the host may affect the progression of carcinogenesis.
8. Fungal dysbiosis can be targeted by the administration of probiotics and prebiotics, fecal microbiota transplantation, and/or antifungal therapies.
